# Prevalence and Determinants of Hepatitis B Antigenemia in 15 007 Inmates in Taiwan

**DOI:** 10.2188/jea.JE20081045

**Published:** 2010-05-05

**Authors:** Ching-Feng Lin, Shiing-Jer Twu, Ping-Ho Chen, Jror-Serk Cheng, Jung-Der Wang

**Affiliations:** 1 Institute of Occupational Medicine and Industrial Hygiene, College of Public Health, National Taiwan University, and Department of Internal Medicine and Department of Environmental and Occupational Medicine, National Taiwan University Hospital, Taipei, Taiwan; 2 Department of Health, Executive Yuan, Taipei, Taiwan; 3 Bali Psychiatric Hospital, Department of Health, Executive Yuan, Taipei, Taiwan; 4 Division of Environmental Health and Occupational Medicine, National Health Research Institutes, Miaoli, Taiwan; 5 College of Public Health, Taipei Medical University, Taipei, Taiwan

**Keywords:** intravenous drug abuse, prisoners, blood transfusion, tattoo, VDRL

## Abstract

**Background:**

The goal of this study was to determine the prevalence and risk factors for horizontal transmission of hepatitis B infection due to intravenous drug abuse (IVDA), tattooing, blood transfusion, and combinations of these risk factors.

**Methods:**

All people detained in 19 prisons were invited to participate. Subjects underwent a physical examination and completed a questionnaire. Blood samples were collected and screened for hepatitis B surface antigen and Venereal Disease Research Laboratory (VDRL) reactivity.

**Results:**

A total of 16 204 prisoners were invited to participate. The response rate was 92.6% and the mean age was 29.73 years. The overall prevalence of HBsAg positivity was 21.7%. Among 3333 subjects with a history of blood transfusion, the carrier rate was 23.19%. Among the 3071 subjects with a history of IVDA and the 6908 subjects with tattoos, the carrier rate was 26.4% and 29.3%, respectively. IVDA appeared to be the strongest risk factor among the 3, with an adjusted odds ratio (AOR) of 1.54 (95% confidence interval, 1.27–1.86), followed by tattooing (1.40, 1.23–1.55), and blood transfusion (1.27, 1.05–1.61). When blood transfusion was combined with either of the other 2 risk factors, the risk increased multiplicatively, and the combination of the 3 factors increased the AOR to 2.76 (2.20–3.47). The prevalence of a positive VDRL test result was 1.01%.

**Conclusions:**

The prevalence of HBV antigenemia in prisoners was high and was associated with BT, IVDA, and tattooing. A national vaccination program against HBV should be considered for prisoners and other people with these risk factors.

## INTRODUCTION

Chronic HBV carriers are a human reservoir, and transmission is known to occur by percutaneous exposures, including blood transfusion, tattooing, surgical procedures, acupuncture, plasma derivative therapy, and by non-percutaneous routes, such as intrafamilial and sexual contact.^1^^,^^2^ Several studies have shown that people with a history of intravenous drug abuse (IVDA) are at high risk of infection with HBV.^3^^–^^5^ However, few studies have investigated the joint effect of more than 2 major risk factors of HBV infection, probably because it is difficult to collect a sufficient number of infected cases.^1^

Taiwan is an endemic area of hepatitis B virus (HBV) infection, with an HBV surface antigen (HBsAg) carrier rate of 15% to 20% in the general population. Approximately 80% to 90% of the adult population has been infected with HBV.^6^^–^^8^ In Taiwan, most HBV HBsAg carriers are infected early in life,^9^^,^^10^ and approximately 40% of HBsAg positivity can be attributed to perinatal transmission.^9^ Beginning in 1984, Taiwan launched a nationwide vaccination program for newborns, which has already decreased the prevalence of HBV infection^11^^,^^12^ and has reduced the incidence rate of hepatocellular carcinoma in young Taiwanese.^13^ However, there has been fewer efforts to control the horizontal transmission routes of HBV in Taiwan. Because IVDA, blood transfusion, and tattooing, or combinations of these risk factors, may still be major routes for the horizontal spread of these viruses, it is important to examine the infection rates of people with a combination of these major risk factors, to ensure future control of hepatitis B. Among prisoners, there is a high prevalence of tattooing, IVDA, and blood transfusions required because of injuries from criminal actions. In addition, the health of prisoners has often been ignored. Thus, to provide information to programs that endeavor to reduce health inequality, we conducted a study among prisoners to explore the relative risks associated with different combinations of these risk factors.

## METHODS

There were a total of 22 prisons with 20 381 prisoners in Taiwan in 1994, when this study was conducted. In 1994, the correctional system of the Ministry of Justice in Taiwan comprised the following facilities: 15 general prisons, 2 detention houses, 2 drug abuse treatment centers, 2 juvenile correctional high schools, and 1 juvenile detention house. All facilities were invited to participate in this study, but 3 general prisons were unable to do so because of lack of adequate manpower.

Although this project was proposed by the CDC (Center for Disease Control) of the Department of Health of Taiwan, it still had to be evaluated by the Justice and Ethical Committee, which included non-medical experts in ethics and law, to ensure protection of the participants’ human rights during the project. This committee was equivalent to the formal IRB (institutional review board) established in 2000 at the Department of Health of the Executive Yuan (the executive branch of the government of Taiwan). A considerable period of time was required to communicate with officials from all these different agencies, and as a result we were only able to successfully include 19 of the 22 prisons when the study was completed at the end of 1994. Thus, under the close supervision and guidance of the Division of Disease Control of the Department of Health, oral informed consent was obtained from every participant during an interview with a public health nurse. A total of 16 204 subjects were invited to participate in this study and 92.6% (15 007) agreed. Five cubic centimeters of blood were drawn from every respondent’s antecubital vein. Because our national newborn vaccination program for HBV was launched in 1985, inmates who were born after 1985 were excluded from the final analysis to prevent confounding.

All blood samples were centrifuged and sera were frozen at −30°C to −70°C until they underwent screening for hepatitis B markers and the Venereal Disease Research Laboratory (VDRL) test. The test for HBsAg was conducted by commercial radioimmunoassay (Abbott Laboratories Diagnosis Division, Abbott Park, IL, USA), according to the manufacturer's instructions. The assay for detection of HBsAg was based on the method first described by Duermeyer et al. It was read with a clinical Gamma Counter (LKB of USA) with both positive and negative controls. If the reading was borderline, the test was repeated 2 or 3 times. If the test was consistently positive, then the result was considered positive. The VDRL test is a blood test for detecting syphilis antibody. Serology was performed by using the T. pallidum hemagglutination assay (TPHA; Fujirebio, Tokyo, Japan), VDRL test (Wellcome Diagnostics, USA), and the fluorescent treponemal antibody absorption (FTA-ABS) test. In the FTA-ABS test, serum antibodies directed against T. pallidum were detected with fluorescein isothiocyanate-labeled horse anti-rabbit immunoglobulin. The sera from 2 infected hares and 4 TPHA-negative, VDRL test-negative, and FTA-ABS test-negative hares were used in this assay at a final concentration of 10% (wt/vol). The percentage of mobile treponemes was determined in wet mounts after 0, 1, 2, 3.5, and 5.5 hours by observing at least 100 treponemes in randomly selected microscopic dark fields.

All serological tests were conducted by the central laboratory of the National Taiwan University Hospital. Every subject was also asked to complete a questionnaire consisting of basic demographic information and previous history of blood transfusion, tattooing, homosexual sex, and intravenous drug abuse. The questionnaire was self-administered with the assistance of public health nurses, who clarified the meaning of words (the illiteracy rate in Taiwan is below 2.8%).^14^ By means of a physical examination, the public health nurses verified every participant with a positive history of tattooing. Because every prisoner was given a complete physical examination and was required to provide 5 to 8 cc of urine for quantitative screening of methylamphetamine, morphine, heroin, marijuana, and MDMA (3,4 methylenedioxymethamphetamine) before entering prison, their history of drug abuse was relatively accurate after verification of their medical records. The nurses also verified a history of blood transfusion by interviewing the prisoners directly.

The data were collected in a computer file and processed and analyzed by using SAS version 6.02. We calculated odds ratios and prevalence odds ratios for HBsAg carrier state using a multivariate logistic regression model, after controlling for major risk factors, including age, gender, and histories of blood transfusion, intravenous drug abuse, and tattooing.

## RESULTS

In total, we successfully collected blood samples and personal information from 15 080 prisoners in 19 of 22 correctional facilities in Taiwan. Three major prisons did not participate in the study because they lacked a sufficient number of qualified health care workers to assist with the study procedures. Excluding these 3 prisons, the participation rate was 92.6%. Among the participants, there were 3333 (22.2%) who had received more than 1 blood transfusion, 3071 (20.5%) who reported intravenous drug abuse, 6908 (46.0%) who had a tattoo, and none who reported having had homosexual sex. There were 73 detainees who were younger than 9 years and were therefore born after 1985, when the national immunization program was implemented; 5 of the 73 were HbsAg-positive and were excluded in the final analysis. The HBsAg carrier rate was approximately 6.8% (5 out of 73 cases) in this subgroup, and none reported blood transfusion, tattooing, or IVDA. After we excluded those aged 9 years or younger, the prevalence of HBsAg was 24.5% (699/2850) among participants aged 10 to 20 years. Approximately 94.4% of the 15 007 inmates were male. The average age was 29.73 ± 9.83 years, and approximately one-third (38.7%) of the detainees were aged 21 to 30 years. Over 40% of prisoners younger than 40 had tattoos, and 25% of prisoners between 30 and 50 years reported IVDA, as shown in Table 1. The prevalence of HBsAg stratified by the various risk factors is also summarized in Table 1. There was a general decreasing trend in HBsAg prevalence after age 30 years, and prevalence among females was 3.5 to 5.7 percentage points lower than prevalence among males. The overall prevalence of HBsAg among the prisoners was 21.7%. The prevalence was 23.2% among those reporting blood transfusion, 26.4% among those reporting IVDA, 29.3% among those with a tattoo, and 12.8% among those without any of these risk factors.

**Table 1.** Frequency distributions and prevalence of HBsAg (%) stratified by demographic characteristics and major risk factors for horizontal transmission in Taiwanese inmates

Major risk factors	No. of subjects	No. of HBsAg(+) carriers/No. with a specific risk factor				VDRL(+)

		Blood Transfusion	Intravenous drug abuse	Tattoo	None^b^	
No. of Cases	15 007	3333	3071	6908	5747	151
		(22.2)	(20.5)	(46.0)	(38.3)	(1.01)

HBsAg(+)	3258	773	810	2022	737	33
	(21.7)	(23.2)^a^	(26.4)^a^	(29.3)^a^	(12.8)	(21.9)
Age (years)						
Mean	29.73	30.47	32.17	27.66	26.72	33.00
SD	9.83	8.83	8.27	8.85	9.12	11.57
10–20	2850	97/445	39/150	290/1238	130/1089	25
		(21.8)	(26.0)	(23.4)	(11.9)	(0.88)
21–30	5815	490/1296	454/1143	1239/3219	345/2224	50
		(37.8)	(39.7)	(38.5)	(15.5)	(0.86)
31–40	3715	195/956	265/1175	366/1667	193/1427	34
		(20.4)	(22.6)	(22.0)	(13.5)	(0.92)
41–50	1588	37/398	55/418	49/506	50/610	25
		(9.3)	(13.2)	(9.7)	(8.2)	(1.57)
≥51	1039	14/238	17/185	17/278	19/397	17
		(5.9)	(9.2)	(6.1)	(4.8)	(1.64)
Sex						
Male	14 167	635/3191	680/2990	1373/6702	713/5425	104
		(19.9)	(22.7)	(20.5)	(13.1)	(0.73)
Female	840	20/122	42/219	24/142	24/322	36
		(16.4)	(19.2)	(16.9)	(7.4)	(4.29)

^a^*P* < 0.001 using crude chi-square test of association between individual risk factors and HBsAg(+).

^b^No blood transfusion, intravenous drug abuse, or tattoo.

VDRL: Venereal disease research laboratory, HBsAg: hepatitis B surface antigen.

There were 151 (1.01%) VDRL-positive detainees. The crude chi-square test revealed that the associations between HBsAg and the individual risk factors of blood transfusion, intravenous drug abuse, and tattooing were all significant (*P* < 0.001); however, there was no such association with VDRL, as shown in Table 1.

Table 2 shows the results of multiple logistic regression analysis, which was adjusted for age, gender, and major risk factors for the horizontal transmission of hepatitis B. Although we added various interaction terms to our model, none was statistically significant. As people grew older, there was a decreased likelihood of being a HBsAg carrier, after controlling for other risk factors. Male detainees were more likely to be carriers than were females (OR, 1.69; 95% confidence interval [CI], 1.21–2.47; prevalence ratio (PR), 1.51; 95% CI, 1.12–2.37). Intravenous drug abuse appeared to be the strongest risk factor among the 3 (OR, 1.54; 1.27–1.86), followed by tattooing and blood transfusion. The odds ratio associated with a combination of blood transfusion and IVDA was 1.72, and that associated with a combination of blood transfusion and tattooing was 1.69, which were both less than what the multiplicative models suggested, namely, 1.27 × 1.54 (= 1.95) is larger than 1.72, and 1.27 × 1.4 (= 1.78) is slightly larger than 1.69. As the combined effect of both risk factors was associated with a higher OR than either one of them alone, the potential of causal interdependence^15^ cannot be ruled out. However, there was no such effect between IVDA and tattooing: the odds ratio associated with these 2 factors was 1.41, which was less than that for IVDA alone (1.54). A combination of the 3 factors resulted in the highest risk—the OR and PR were 2.76 (95% CI, 2.20–3.47) and 2.30 (1.71–3.05), respectively.

**Table 2.** Adjusted odds ratios (aORs) and prevalence ratios (aPRs) with 95% CIs (confidence intervals) calculated using a multivariate regression model adjusted for age, gender, and major risk factors for horizontal transmission of Hepatitis B

Risk factor			No. of HBsAg carriers/total no.	Crude prevalence	aOR (95% CI)	aPR (95% CI)
Total			3258/15 007	21.7%		
Age (years)						
10–20			699/2850	24.5%	1.0	1.0

21–30			1299/5815	22.3%	0.93 (0.81–1.05)	0.94 (0.83–1.05)
31–40			778/3715	20.9%	0.84 (0.71–0.95)	0.85 (0.72–0.97)
41–50			295/1588	18.6%	0.64 (0.49–0.76)	0.65 (0.53–0.84)
≥51			187/1039	18.0%	0.45 (0.34–0.64)	0.49 (0.36–0.69)
Sex (male/female)			3109/14 167	21.9%	1.69 (1.21–2.47)	1.51 (1.12–2.37)
			149/840	17.6%		
Exposure:						
Blood Transfusion	IVDA	Tattoo				
−	−	−	737/5747	12.8%	1.0	1.0
+	−	−	266/1601	16.6%	1.27 (1.05–1.61)	1.22 (0.97–1.53)
−	+	−	191/917	20.8%	1.54 (1.27–1.86)	1.45 (1.17–1.80)
−	−	+	1070/3718	28.8%	1.40 (1.23–1.55)	1.33 (1.25–1.51)
+	+	−	77/230	33.5%	1.72 (1.57–1.97)	1.60 (1.39–1.89)
+	−	+	408/1105	36.9%	1.69 (1.45–2.05)	1.57 (1.28–1.91)
−	+	+	392/1686	23.3%	1.41 (1.21–1.64)	1.35 (1.13–1.59)
+	+	+	154/401	38.4%	2.76 (2.20–3.47)	2.30 (1.71–3.05)

IVDA: intravenous drug abuse.

+: exposure, −: non-exposure.

## DISCUSSION

We examined the prevalence rates of HBsAg in a very large sample, in which more than 3000 participants had at least 1 of the risk factors of interest. Because all 352 subjects in the 2 drug abuse treatment centers were included in this study, the proportion of detainees with IVDA might be overestimated; however, this does not seem to have affected the estimated prevalence rate for HBsAg in this subgroup. The possibility of horizontal transmission was suggested in the present study by the fact that HBsAg carrier prevalence among prisoners with a history of BT, IVDA, or tattooing was higher than 23%, which was significantly higher than both the prevalence for prisoners without any of such exposures (12.8%) and that noted in previous general population surveys (18.7%).^6^ None of our subjects revealed a history of homosexual sex, as it was still a social taboo at the time of the survey. Because the prevalence of homosexuality is low in Taiwan (2% to 3%),^16^ and there is no association between criminal activity and homosexuality,^17^ the likelihood of transmission of HBV through homosexual sex among inmates is probably low, and any potential confounding of the estimated prevalence rates by homosexuality is likely to be minimal in this study. The risk for transmission by blood transfusion (AOR, 1.27) was the lowest among the 3 factors. This may be due to a national policy established in 1984, which required that all blood products intended for medical use must be screened and confirmed to be HBV-negative. IVDA and tattooing were verified by a nurse by means of medical records and physical examination for every subject indicating such a history. Due to the possibility of recall bias, we did not ask for the dates of blood transfusion and therefore cannot stratify subjects by date of blood transfusion (ie, before or after the implementation of the blood-screening program in 1984). This may have led to an underestimation of the overall OR for HbsAg positivity associated with blood transfusion.

We attempted to add different interaction terms to our model, but none of the combinations of 2 or 3 terms was statistically significant, and were thus not included in the final model shown in Table 2. However, since a logistic regression model essentially assumes a multiplicative effect between different risk factors, an insignificant finding for an interaction term does not rule out additive interaction—which may have important public health implications^18^—or causal interdependence.^15^ Because the combinations of blood transfusion and either IVDA or tattoo, and the combination of all 3 of these factors, had a roughly multiplicative effect (namely, 1.54 × 1.4 × 1.27 = 2.74, which is similar to our actual estimate of 2.76), we believe that there is an interaction effect. However, although tattooing and IVDA increased the odds of HBsAg-positivity to 1.40 and 1.54, respectively, the combination of both factors only increased the OR to 1.41. One of the most plausible explanations for this result is that people sharing a common needle for either tattooing or drug use were usually from the same homogeneous group. Indeed, 37.3% of the 3071 subjects in the IVDA population had a tattoo, and 34.3% of the 6908 subjects with a tattoo were positive for IVDA. Thus, repeated exposures can infect only the maximum proportion of susceptible individuals. An alternative explanation is that hepatitis C virus infection might suppress HBV activity, thereby resulting in a negative reaction to the usual HBsAg marker.^19^^–^^23^ The odds of disease were 1.72 times higher for subjects with a combined history of BT and IVDA, as compared with those with a history of neither, which is consistent with the findings of a study on the combined risk of combined BT and acupuncture.^1^ The odds ratio of hepatitis B among subjects with exposures via all 3 major horizontal routes was 2.76 times that of participants with none, indicating a multiplicative effect. Thus, we conclude that the hepatitis B virus can be independently and effectively transmitted through these 3 routes and that combined exposures of any risk factor(s) with blood transfusion further increases the risk, which should be carefully considered in future programs for hepatitis B infection control that are due to be undertaken after the national vaccination program.^11^^–^^13^ In other words, careful screening of blood products for hepatitis B must be emphasized. To reduce health inequality, intensive health education and a voluntary vaccination program covered by the NHI (National Health Insurance) is recommended for detainees in correctional facilities in Taiwan.

To our surprise, the prevalence of VDRL seroreactivity was only 1.01%. Although the frequency of blood transmission exposures was high, with more than 64.4% (*n* = 9658) exposed to at least 1 procedure, the prevalence of syphilis did not significantly differ from that of the general population.^24^ One reason for this may be that, in Taiwan, there is routine screening for VDRL and regular treatment for syphilis when an inmate is admitted. However, the reason for the difference in prevalence between males and females deserves further study. A plausible explanation is that treatment of syphilis in female inmates was less comprehensively enforced, which may have resulted in a local endemic that would need to be addressed. Because our sample size was large, we tentatively conclude that the blood transmission route is not effective in transmitting syphilis, although there have been occasional case reports that suggest otherwise.^25^ Future occupational health guidelines might thus be able to exclude VDRL testing for needle stick injuries, unless the contaminated blood comes from a patient with a history of syphilis.

Taiwan has the highest average ratio of motorcycles per person in Asia. In 2008, there were more than 12 million motorcycles in Taiwan. The prevalence of traffic accidents involving motorcycles was very high over a recent 10-year period, a fact which is related to the high prevalence of blood transfusions. The prevalence rate for HBsAg among all inmates with a history of blood transfusion was 23.19%; it was 26.38% among all those with a history of intravenous drug abuse. However, among inmates aged between 10 and 20 years, the respective prevalence rates were 15.6% and 5.3%. Because Taiwanese teenagers are allowed to ride motorcycles after age 18 years and many suffer motorcycle injuries that may require blood transfusions, the prevalence of blood transfusions increased to 22.3% by age 30 years, but none of the 73 detainees younger than 9 years had a history of blood transfusion. With increasing age, the likelihood of HBsAg positivity decreased (Table 2). This can be explained by the increased mortality of older HBsAg carriers due to liver cirrhosis and/or cancer, and by the effect of increased HBsAg seroclearance in asymptomatic adult carriers.^26^^,^^27^

In conclusion, the present study shows that BT, tattooing, and IVDA contributed to a significant proportion of the load of hepatitis B antigenemia in the entire sample, and that there seems to be a potential public health interaction effect among different combinations of the risk factors for horizontal transmission. A vaccination program for HBV should thus be considered for prisoners.
